# Prevalence of Asthma among Norwegian Elite Athletes

**DOI:** 10.1155/2022/3887471

**Published:** 2022-07-06

**Authors:** Helene Støle Melsom, Anders Randa, Jonny Hisdal, Julie Sørbø Stang, Trine Stensrud

**Affiliations:** ^1^Institute of Clinical Medicine, Faculty of Medicine, University of Oslo, Oslo, Norway; ^2^Department of Vascular Surgery, Oslo University Hospital, Oslo, Norway; ^3^Department of Sports Medicine, Norwegian School of Sport Sciences, Oslo, Norway

## Abstract

**Objective:**

Asthma is a common problem among elite athletes and represents a health risk interfering with the athlete's performance status. This study aimed to evaluate the asthma prevalence among Norwegian summer and winter elite athletes and asthma prevalence across sport categories. We also aimed to examine whether bronchial hyperresponsiveness (BHR), lung function, fraction of exhaled nitric oxide (FE_NO_), and allergy status differed between asthmatic and non-asthmatic elite athletes.

**Methods:**

Norwegian athletes qualifying for the Beijing Olympic Summer Games 2008 (*n* = 80) and the Vancouver Olympic Winter Games 2010 (*n* = 55) were included. The athletes underwent clinical respiratory examination including lung function measurement, methacholine bronchial challenge for assessment of BHR, FE_NO_, and skin prick testing. Asthma was diagnosed based on respiratory symptoms and clinical examination including objective measurements.

**Results:**

Asthma was more prevalent among winter athletes (50%) than summer athletes (20%). Thirty-three (52%) endurance athletes, 3 (6%) team sport athletes, and 7 (33%) technical sport athletes had medically diagnosed asthma. Significantly lower lung function (*p* < 0.001) and higher prevalence of severe BHR (*p* < 0.001) were found in asthmatic athletes compared with non-asthmatic athletes.

**Conclusion:**

Asthma is common among Norwegian elite athletes, with winter and endurance athletes showing the highest prevalence. Asthmatic athletes were characterized by lower lung function and more severe BHR compared with non-asthmatic counterparts. The high prevalence among winter and endurance athletes demonstrates a need for increased attention to prevent and reduce the prevalence of asthma among those athletes.

## 1. Introduction

A diagnosis of asthma is more frequent among elite endurance athletes than both the general population and athletes within other types of sport [1, 2]. Asthma is characterized by chronic airway inflammation and bronchial hyperresponsiveness (BHR), which increased sensitivity to a wide variety of airway narrowing stimuli [1]. The reported prevalence varies between studies [3–6] and type of sport [4, 7]. The diagnosis of asthma should be based on objective measurements, in addition to symptoms and clinical examination [1]. The reported asthma prevalence among athletes varies with the criteria used for diagnosis: questionnaires, anti-doping records, spirometry, or bronchial provocation challenges [8]. However, asthma is a significant problem among elite athletes and represents both a health risk and can interfere with the athlete's performance status [9].

Endurance athletes may be at greater risk of developing asthma, possibly due to higher ventilation rates over prolonged periods of time, combined with unfavorable sport-specific environmental conditions, such as cold and dry air, indoor swimming pools, and polluted air [1, 10]. Some investigators also suggest a difference between summer and winter sports, with the highest asthma prevalence observed in winter sports [7]. Asthma prevalence in Olympic summer and winter athletes has been compared in a small number of questionnaire-based studies [4, 11, 12]. Weiler and Ryan found that more winter athletes than summer athletes suffered from asthma [11, 12]. This contrasts with the study by Selge et al. [4] of German athletes competing in 2008 Beijing Olympic Summer Games and 2010 Vancouver Olympic Winter Games. They found no difference in asthma prevalence between winter and summer athletes [4]. However, they observed that type of sport was associated with the asthma prevalence.

Athletes seem to be more affected by the asthma phenotype called sports asthma. This asthma phenotype is characterized by BHR to exercise that usually occurs after the cessation of a short period of hyperpnea and lasts for about 30–90 minutes when not treated [1, 13]. Characteristics of sports asthma include late onset (in early adulthood) and symptoms such as cough, phlegm, and heavy breathing in response to physical activity [14, 15].

The scientific interest of exercise-induced bronchoconstriction (EIB) can be dated back to the 1960s, when Jones and coworkers focused on physiologic responses to exercise in asthmatic children [16]. Since then, research on asthma prevalence in athletes has grown, as well as research on the mechanisms of the development of the phenotype: sports asthma [8]. Carlsen [17] suggested that the mechanisms of sports asthma are a combination of respiratory epithelial damage caused by regularly repeated bouts of increased ventilation in cold and polluted air and increased parasympathetic tone [17]. Together, this may result in bronchial hyperresponsiveness and asthma symptoms [17].

As asthma is a significant health problem among elite athletes, the need for greater knowledge about athletes' respiratory health is warranted. Many studies on asthma prevalence in athletes are based on questionnaires [1], which is not in accordance with current guidelines from Global Initiative of Asthma (GINA) or recommendations from the International Olympic Committee—Medical Commission (IOC-MC) on diagnosing asthma [18]. Hence, the primary aim of this study was to evaluate the prevalence of asthma in Norwegian summer and winter elite athletes and across sport categories (endurance, team sport, and technical sport) using objective measurements in addition to clinical examination. Secondly, we aimed to assess whether BHR, fraction of exhaled nitric oxide (FE_NO_), allergy, and lung function differed between asthmatic and non-asthmatic elite athletes.

## 2. Methods

The study was carried out as part of a multicenter study: “The Asthma and Allergy in Olympians Study.” This was conducted within the framework of the Global Allergy and Asthma European Network (GA^2^LEN) across nine European countries. This study includes cross-sectional data of 135 Norwegian athletes aiming to qualify for the Beijing Olympic Summer Games 2008 and the Vancouver Olympic Winter Games 2010 within different sports. All participants underwent clinical respiratory examination including lung function measurement, measurement of FE_NO_, allergy assessment with a skin prick test (SPT), and a methacholine bronchial challenge (PD_20met_) for the assessment of bronchial hyperresponsiveness (BHR). The clinical examination was performed in one day at the respiratory laboratory at the Norwegian School of Sport Sciences by the same doctor and respiratory physiologist.

Asthma medication was withheld prior to testing according to current guidelines given by European Respiratory Society (ERS) [19]. Inhaled short-acting beta-2 agonists were withheld for 8 h prior to testing, inhaled long-acting beta-2 agonists, theophylline, and leukotriene antagonists for the preceding 72 h, antihistamines for 7 days, and orally administered glucocorticoids for the preceding 3 months. On the test day, inhaled glucocorticoids were withheld and participants were instructed to refrain from exercise and food or drinks containing nitrate.

### 2.1. Definitions

Current asthma was defined as a doctor's diagnosis of asthma. The diagnosis was set by the doctor at the Norwegian School of Sport Sciences. This diagnosis was based on clinical examination, reported symptoms, lung function measurements, and bronchial hyperresponsiveness (BHR) to methacholine (PD_20met_). PD_20met_ was defined as the dose of methacholine causing a 20% reduction in forced expiratory volume in one second (FEV_1_). Bronchial hyperresponsiveness was defined as PD_20met_ ≤8 *μ*mol. The diagnosis could also be verified by demonstration of exercise-induced bronchoconstriction (EIB test) or an increase in forced expiratory volume in one second (FEV_1_) of ≥12% after administration of short-acting beta-agonists (SABAs) or short-acting muscarinergic agonists (SAMAs).

### 2.2. Design and Subjects

All subjects gave their written informed consent for participation. The study was approved by the Regional Committee for Medical and Health Research Ethics South East Norway (REC; Ref: S-07468a).

In this study, 80 Norwegian summer athletes qualifying for the 2008 Olympic Summer Games in Beijing and 55 Norwegian winter athletes qualifying for the 2010 Olympic Winter Games in Vancouver are included. Characteristics are presented in Table 1. All athletes were categorized into endurance, technical and team sports, and Summer or Winter Games. Endurance sports include swimming, cycling, rowing/paddling, long-distance running, cross-country skiing, biathlon, speed skating, and Nordic combined. Both males and females were included in all endurance sports except for Nordic combined where only males are included. Only 21 athletes are included in the technical sports group; therefore, types of sports or number of males/females are not reported to maintain anonymity. The team sports group consists of summer athletes only.

### 2.3. Lung Function

Lung function was measured by maximal expiratory flow-volume curves (MasterScreen Pneumo Jäger ®, Würzburg, Germany) according to current guidelines [20] and recorded as FEV_1_, forced vital capacity (FVC), and mean expiratory flow between 25 and 75% of FVC (MEF_25–75_). Predicted values are according to the last updated reference values from Global Lung Function Initiative (GLI 2012) published by Quanjer et al. [21]. FEV_1_, FVC, and MEF_25–75_ are reported as percentages predicted and Z-scores. Z-scores less than −1.645 were considered below the lower limit of normal (LLN).

### 2.4. Bronchial Hyperresponsiveness

Bronchial hyperresponsiveness was performed by methacholine bronchial provocation. Methacholine was delivered by an inspiratory-triggered nebulizer aerosol provocation system (Jäger Würzburg, Germany). It was inhaled in doubling doses from a starting dose of 0.25 *μ*mol until FEV_1_ decreased ≥20% from baseline or if the maximal dose of methacholine (24.48 *μ*mol or 4.8 mg) was reached, as measured after inhaled nebulized isotonic saline. The dose causing 20% reduction in FEV_1_ (PD_20met_) was determined by linear interpolation on the semi-logarithmic dose-response curve. A subject was considered to have BHR if their PD_20met_ was ≤8 *μ*mol (1.6 mg).

### 2.5. Expired Nitric Oxide

FE_NO_ was measured with a single-breath technique at a constant expiratory flow rate of 50 mL/s in accordance with the manufacturer's instructions (Eco Medics AG, Dürnten, Switzerland) and American Thoracic Society (ATS)/ETS Guidelines [22]. Mean values of three measurements with <10% difference were used in the analysis.

### 2.6. Skin Prick Test

Local allergic sensitivity was assessed according to Nordic guidelines [23] with the following allergens: moulds (Cladosporium herbarum), house dust mites (Dermatophagoides pteronyssinus), dog dander, cat dander, birch pollen, grass pollen (Timothy), mug worth pollen, milk, shrimp, and hen's egg white (Soluprick, ALK, Copenhagen, Denmark). A positive response was defined if the weal was at least ½ of the histamine (10 mg·mL^−1^) reaction or if the average weal exceeded 3 mm.

### 2.7. Analyses

Continuous data are presented as means with standard deviation (SD) or 95% confidence intervals (CIs) after the Shapiro–Wilk test for normality unless otherwise stated. Categorical variables are presented as counts (*n*) with percentages (%). One-way analysis of variance (ANOVA) with the Bonferroni post hoc tests or, in the case of nonparametric data, the Kruskal–Wallis tests were used to compare the three groups. The Mann–Whitney *U* test for independent samples was used to compare two groups of nonnormally distributed data. Chi-square tests were used to assess group differences of categorical variables. Sex differences for asthma and allergy prevalence, as well as BHR, were only tested for all included athletes and within the groups of summer and winter athletes. Due to small sample size, we could not test sex differences among the athletes in the sport categories. *p* values below 0.05 were considered significant. Statistical analyses were performed using IBM SPSS Statistics version 26 (SPSS Inc., Chicago, IL, USA) and SigmaPlot 14.5 (Systat Software, San Jose, CA).

## 3. Results

### 3.1. Study Population

In total, 135 athletes were included in the study, 80 **(♀** **=** **62♂** **=** **18)** summer athletes and 55 **(♀** **=** **20♂** **=** **35)** winter athletes. Characteristics are shown in Table 1.

### 3.2. Clinical Measurements

**Figure 1:**
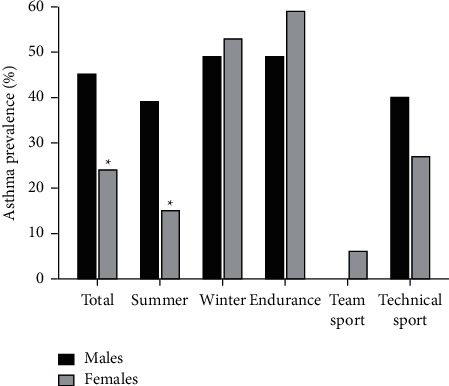
Differences in asthma prevalence between men and women, divided into summer and winter athletes, and by sport category.

Of the 135 Norwegian athletes, 16 of 80 summer athletes (20%) and 27 of 55 winter athletes (50%) had medically diagnosed asthma (*p* < 0.001, Table 2). Thirty-three (52%) endurance, 3 (6%) team sport, and 7 (33%) technical sport athletes had medically diagnosed asthma (*p* < 0.001, Table 2). We observed significantly higher prevalence of asthma (45%) among males compared with females (23%) (*p*=0.008) for all included athletes and among males (39%) compared with females (15%) competing in summer Olympics (*p*=0.041). However, no sex difference was seen among the winter athletes, males (49%) and females (53%), respectively (*p*=1.000, Figure 1). Within the endurance group, 20 of 41 males (49%) and 13 of 22 females (59%) had a diagnosed asthma (Figure 1).

Allergy status did not differ between summer and winter athletes (*p*=0.166), nor when dividing the athletes into different sport categories (*p*=0.403, Table 2). Allergy prevalence was the same in athletes with asthma (*n* = 18; 41.9%) as in athletes without asthma (*n* = 34; 39.1%, *p*=0.761, Table 3). No sex difference was seen for allergy prevalence for all included athletes (*p*=0.097), either among summer athletes (*p*=0.557) or among winter athletes (*p*=0.390).

### 3.3. Lung Function

**Figure 2:**
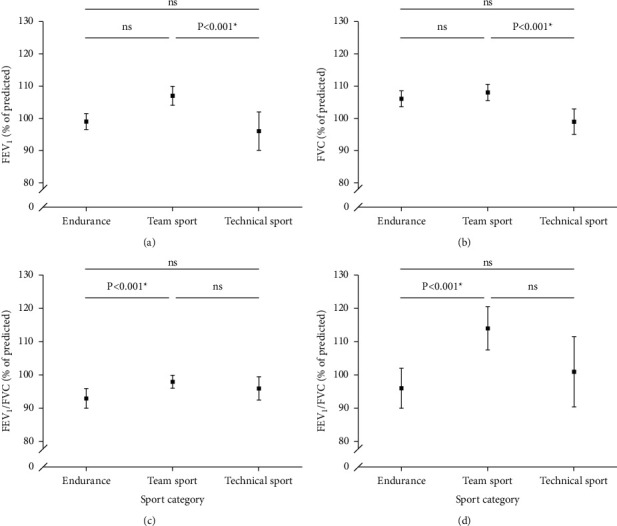
Lung function, forced expiratory volume in one second (FEV_1_), forced vital capacity (FVC), mean expiratory flow between 25 and 75% of FVC (MEF_25–75_), and FEV_1_/FVC in elite athletes across sport categories. ns = not significant different (*p* > 0.05); FEV1 = forced expiratory volume in one second; FVC = forced vital capacity; MEF25-75 = mean expiratory flow between 25 and 75% of FVC.

Summer athletes displayed significantly better lung function (FEV_1_, FVC, MEF_25–75_, FEV_1_/FVC) both as percentage of predicted parameters and higher *Z*-scores than winter athletes (Table 4). When comparing the different sport categories, lung function differed significantly between groups (Figure 2), both in % of predicted and in *Z*-scores (*p* < 0.001, Table 3).

Bronchial hyperresponsiveness was less prevalent in summer athletes than in winter athletes ((*n* = 23; 29%) vs 29; 53%, *p*=0.005, Table 2). Furthermore, BHR differed significantly between sport categories (*p*=0.041), with the highest prevalence in endurance athletes (*n* = 34; 53%) compared with team sport (*n* = 9; 18%) and athletes in technical sports (*n* = 10; 50%; Table 2). We did not observe sex differences for BHR (PD_20met_ ≤8 **μ**mol) among all included athletes (*p*=0.106), either among summer athletes (*p*=0.375) or among winter athletes (*p*=1.000).

There was no difference in FE_NO_ between summer and winter athletes (*p*=0.933), nor between the different sport categories (*p*=0.867, Table 3).

## 4. Discussion

In this study, we assessed the prevalence of asthma among Norwegian elite winter and summer athletes using standardized protocols. Our main finding is that asthma is more prevalent in Norwegian elite winter athletes (50%) than in summer athletes (20%), which is considerably higher than in the general population (8–10% in adolescents) [18, 24]. In addition, the characteristics of asthmatic elite athletes included more severe BHR and reduced lung function compared with non-asthmatic elite athletes.

We found asthma to be less prevalent among Norwegian elite summer athletes than among their winter counterparts. This is in line with the findings from two studies by Weiler and Ryan, where they studied asthma prevalence in 699 summer and 196 winter athletes [11, 12]. They found asthma to be less prevalent among US summer athletes during the 1996 Olympic summer games than among US winter athletes during the 1998 Olympic Winter Games. In these studies, the prevalence was found to be 15% among summer athletes and 22% among winter athletes, lower than that found in our study (20% and 50%, respectively). Although the results from our study and the studies by Weiler et al. are partly in agreement, the studies by Weiler et al. were questionnaire-based. A strength of our work and possible reason for this discrepancy may lie in our data that are based on objective clinical measurements.

In contrast, a study by Selge et al., examining German Olympic summer (*n* = 283) and winter (*n* = 265) athletes, examined the prevalence of medically diagnosed asthma [4]. They found no association between asthma prevalence and competition season (17.1% vs 12.1%) [4], which conflicts with our results and the study by Weiler et al. These findings might reflect that the studies are based on differing methods and sample sizes. However, it is remarkable that we found the asthma prevalence to be as high as 20% in the Norwegian summer athletes and 50% in the winter athletes, while results from the other studies show a considerably lower prevalence. Winter athletes exercise and compete in cold and dry air, which might be harmful to the airways [25]. However, there are also studies demonstrating a high asthma prevalence in summer athletes [2, 12], with swimming, long-distance running, and road cycling being examples of high-prevalence sports [14, 26, 27].

When dividing the sports into different categories depending on the ventilation rate during competition, we found that sports with high ventilation rates (endurance sports) have a higher prevalence of asthma compared with technical sports and team sports. This is in line with previous studies [4, 10, 28]. Results from this study show that endurance athletes are at increased risk of developing asthma. Results from previous studies show that asthma is a problem among swimmers, as well as cyclists, long-distance runners, and cross-country skiers [3, 5, 6, 27, 29]. Bougault et al. studied the airways of 32 swimmers and 32 cold air athletes (11 speed skaters, 16 cross-country skiers, and 5 biathletes) [26]. Airway hyperresponsiveness was found in 69% of the swimmers and 28% of the cold air athletes [26]. These results indicate that endurance athletes in both summer sports and winter sports are at increased risk of developing asthma, due to unfavorable environmental conditions. Winter athletes are exposed to large amounts of cold air, summer athletes to allergens, and swimmers to chlorine and its derivatives [30]. Thus, endurance athletes are exposed to various factors that affect air quality and contribute to the development of asthma.

We found a significantly higher prevalence of asthma in males compared with females competing in the summer Olympics, but not in the winter Olympics (Figure 2). No difference was observed for allergy prevalence and BHR between sexes. Langdeau et al. [31] reported that BHR, assessed with methacholine challenge, was significantly higher in female athletes compared with male athletes and that allergy assessed by one or more positive SPT was more prevalent among males. However, physician-diagnosed asthma (self-reported) was similar between sexes [31]. This is not in accordance with our results and could probably be explained by the large female ball game group participating in the summer Olympics. They were least affected with asthma, BHR, and allergy (15%, 26%, and 33%, respectively), compared with the males (39%, 39%, and 44%, respectively). Due to the small sample size in the sport groups, we cannot test for sex differences and can only suggest that it seems like asthma prevalence that is similar between sexes (Figure 1).

During exercise, ventilation increases considerably, sometimes reaching more than 200 L/min [32]. Ventilation at these levels in cold, dry, and polluted air exposes athlete's airways to frequent unfavorable conditions over time [27, 30]. Increased ventilation also causes heat and water loss from the airway epithelium due to insufficient warming and humidification of the inhaled air. Insufficient warming and humidification result in heat transfer from the mucosa into the inspired, cold air, which causes a vasoconstriction of the microvasculature within the airway. This is followed by a subsequent rebound hyperemia after exercise when the airway rewarms, which results in vascular leakage and edema resulting in airway narrowing [33]. The loss of water leads to a change in airway osmolarity that initiates epithelial cell and mast cell activation, leading to release of inflammatory mediators in the airways that cause bronchoconstriction [34, 35]. Reduced warming and humidification of inspired air happen when switching from nasal breathing to mouth breathing when ventilation levels reach about 30 L/min [27, 36]. The shift from nasal breathing to mouth breathing also leads to incomplete filtering of the air reaching the lower airways [27]. The combination of insufficient air warming and less air causes airway epithelium damage, and thus contribute to the development of asthma [37].

Despite the difference in asthma prevalence and lung function between summer and winter athletes, and between the sport categories, we did not observe any significant difference in FE_NO_ between those with medically diagnosed asthma or between the sport categories. FE_NO_ is among the biomarkers used to assess airway inflammation. However, the evidence is divergent. High levels of FE_NO_ do not necessarily reflect the severity of asthma or asthma control/lung function parameters [38], especially in relation to sports asthma [14]. This is in line with results from a meta-analysis by Mäki-Heikkila et al., where no difference was found in FE_NO_ and inflammatory markers in induced sputum between the asthmatic skiers and healthy controls [39]. Neither was FE_NO_ different between skiers and healthy controls and was lower in skiers than in asthmatic controls [39]. These results suggest that the distribution of inflammatory endotypes may differ between skiers and nonskiers with asthma. Furthermore, results indicate that eosinophilic inflammation may not be as prevalent in skiers with asthma and that skiing even in the absence of asthma may trigger non-eosinophilic inflammation. However, the low levels of FE_NO_ observed in this study support the theory that sports asthma has a different pathology to atopic asthma. This may indicate that the gold standard treatment with ICS according to GINA is not optimal for sports asthma. However, more research on the relationships between asthma phenotypes, airway inflammation, and treatment of sport asthma is needed. Even if FE_NO_ is not expected to be elevated in athletes with asthma, it could be used as a complementary tool to avoid unnecessary increases in anti-inflammatory medication. We found no difference in allergy prevalence across the groups, nor between asthmatic athletes and non-asthmatic athletes. These results further support the theory that sports asthma is driven by other factors than allergy [14, 17], such as increased parasympathetic activity [40].

When dividing all athletes into asthmatic and non-asthmatic groups, we found baseline lung function to be significantly lower in asthmatic athletes compared with non-asthmatic athletes.

All groups' mean lung function was within normal range, with summer athletes and team sport athletes having the highest lung function. This is in line with previous studies that have shown high lung volumes and high airflow rates in swimmers [41, 42]. Our results also suggest that team sport athletes have higher lung function. In this study, all team athletes were ball game players and almost all were female. Rosser-Stanford et al. have similarly suggested better than average lung function in this group [42]. Both swimmers and team sport players represent summer athletes, which may partly explain why summer athletes have higher lung function than winter athletes.

Particular strengths of this study were the use of objective measures of lung function, BHR, and allergy testing to diagnose asthma in both summer and winter top-level athletes aiming to qualify for the Olympic games. Furthermore, all measurements were performed by the same trained personnel. Nevertheless, the results should be interpreted with some considerations in mind. All team sport athletes were ball game players and almost only women; therefore, our results may be affected with the inclusion of more male participants. Bronchial hyperresponsiveness (BHR) was measured using methacholine provocation test; however, eucapnic voluntary hyperpnea (EVH) can be considered the gold standard [43]. The cross-sectional design of this study also limits the possibility to infer causality. Therefore, we emphasize the need for and importance of prospective studies to examine the development of respiratory disorders among athletes. Although athletes were instructed to avoid inhaled corticosteroids (ICS) one day prior to testing, the effects of these medications were potentially still present in those who used them, which may have influenced our results.

## 5. Perspectives

This study shows that Norwegian elite athletes have high asthma prevalence and reduced lung function, particularly among winter athletes and those involved in endurance sports. The characteristics of asthmatic elite athletes were more severe BHR and reduced lung function compared with non-asthmatic athletes. Allergy prevalence and FE_NO_ >25 ppb did not differ significantly between asthmatic and non-asthmatic elite athletes. Athletes, medical staff, and coaches should be aware of the high asthma prevalence among athletes training in unfavorable environmental conditions such as cold and dry air, polluted air, and swimming pools with high concentration of chlorine combined with poor ventilation. However, it is still unclear whether asthma, BHR, and reduced lung function are persistent in elite athletes after retirement from elite sport and whether respiratory health may be a limitation for physical activity and exercise after career end. Follow-up studies are needed to evaluate how athletes' respiratory health is over time and whether the asthma diagnosis is reversible.

## Figures and Tables

**Table 1:** Characteristics of elite athletes, divided into summer and winter athletes and across sport categories. Results are presented as mean and standard deviation (SD) and sex differences as number and percentage (%).

Variables	Competition season			Sport categories			
	Summer (*n* = 80) mean (SD)	Winter (*n* = 55) mean (SD)	*p* value	Endurance (*n* = 64) mean (SD)	Team sport (*n* = 50) mean (SD)	Technical (*n* = 21) mean (SD)	*p* value
Age (years)	27 (4.5)	26 (6.1)	0.564	26 (5.0)	27 (4.3)	30 (6.8)	0.016
Height (cm)	176 (8.3)	178 (8.2)	0.953	179 (8.2)	175 (7.8)	176 (8.1)	0.014
Weight (kg)	71 (10.7)	74 (11.4)	0.391	74 (10.8)	69 (8.4)	73 (15.8)	0.054
Sex (♀), *N* (%)	62 (78)	20 (36)	<0.001^*∗*^	23 (36)	48 (96)	11 (52)	<0.001

**Table 2:** Prevalence of medically diagnosed asthma, bronchial hyperresponsiveness (BHR), and allergy defined as one or more positive skin prick tests (SPT) among elite athletes, divided into summer and winter athletes and across sport categories.

	Summer (*n* = 80)	Winter (*n* = 55)	*p* value	Endurance (*n* = 64)	Team sport (*n* = 50)	Technical (*n* = 21)	*p* value
Asthma *N* (%)	16 (20)	27 (50)	<0.001	33 (52)	3 (6)	7 (33)	<0.001
^#^BHR *N* (%)	23 (29)	29 (53)	0.005	34 (53)	9 (18)	9 (43)	0.041
^*∗*^Allergy *N* (%)	27 (35)	25 (47)	0.166	26 (41)	16 (33)	10 (50)	0.403

BHR is defined as PD_20met_ ≤8 *μ*mol. PD_20met_: provocation dose of methacholine causing 20% reduction in forced expiratory volume in the first second of expiration. ^#^: for doctor-diagnosed asthma, 54 winter athletes and 63 endurance athletes are included; ^*∗*^: for allergy assessment, 77 summer athletes, 53 winter athletes, 62 endurance athletes, 48 team sport athletes, and 20 technical sport athletes are included.

**Table 3:** Bronchial hyperresponsiveness measured as PD20met <8 *μ*mol, fractional expired nitric oxide FENO >25 ppb, allergy and lung function variables presented as *Z*-score, and lower limit of normal (LLN) among elite athletes with doctor-diagnosed asthma and elite athletes without asthma.

Variables	Current asthma		*p* value
	Yes (*n* = 43)	No (*n* = 90; 87; 91)^#^	
PD_20met_ <8 *μ*mol, *n* (%)	37 (86)	14 (15)	<0.001^*∗*^
FE_NO_ >25 ppb, *n* (%)	11 (26)	11 (12)	0.079
Allergy, *n* (%)	18 (42)	34 (39)	0.850
z-FEV_1,_ mean (95% CI)	−0.50 (−0.78, −0.22)	0.42 (0.25, 0.62)	<0.001^*∗*^
z-FVC, mean (95% CI)	0.34 (0.04, 0.63)	0.50 (0.35, 0.68)	0.288
z-MEF_25–75,_ mean (95% CI)	−0.53 (−0.85, −0.20)	0.45 (0.25, 0.64)	<0.001^*∗*^
z-FEV_1_/FVC, mean (95% CI)	−1.24 (−1.50, −0.97)	−0.21 (−0.38, −0.03)	<0.001^*∗*^
LLN FEV_1_*n* (%)	4 (9)	0 (0)	0.010^*∗*^
LLN FVC *n* (%)	0 (0)	0 (0)	ns
LLN MEF_25–75_*n* (%)	5 (12)	0 (0)	0.003^*∗*^
LLN FEV_1_/FVC *n* (%)	13 (30)	3 (3)	<0.001^*∗*^

PD_20met_ = provocation dose of methacholine causing 20% reduction in forced expiratory volume in the first second of expiration; allergy is defined as one or more positive skin prick tests; FEV_1_ = forced expiratory volume in one second; FVC = forced vital capacity; MEF_25–75_ = mean expiratory flow between 25 and 75% of FVC; LLN = lower level of normal defined as *Z*-score <−1.645. ^#^ = for the no asthma group; *n* = 89 for FE_NO_, *n* = 87 for allergy, and *n* = 91 for LLN and lung function values ns = not significant ^*∗*^ = significant difference between asthmatic and non-asthmatic athletes.

**Table 4:** Lung function and fractional expired nitric oxide (FE_NO_) among elite athletes, divided into summer and winter athletes and across sport categories.

		Summer (*n* = 80) mean (95% CI)	Winter (*n* = 55) mean (95% CI)	*p* value	Endurance (*n* = 64) mean (95% CI)	Team sport (*n* = 50) mean (95% CI)	Technical (*n* = 21) mean (95% CI)	*p* value
FEV_1_	(% pred)	105 (103, 108)	96 (93, 99)	<0.001	99 (96, 101)	107 (104, 110)	96 (90, 102)	<0.001
	z-FEV_1_	0.45 (0.26, 0.65)	−0.33 (−0.58, −0.08)	<0.001	−0.11 (−0.33, 0.11)	0.63 (0.37, 0.38)	−0.33 (−0.80, 0.15)	<0.001
	LLN (*n*, %)	0 (0%)	4 (7.4%)	0.026	2 (3.2%)	0 (0%)	2 (9.5%)	<0.001

FVC	(% pred)	108 (106, 111)	102 (100, 105)	0.001	106 (103, 108)	108 (106, 111)	99 (95, 103)	<0.001
	z-FVC	0.66 (0.47, 0.84)	0.19 (−0.0, 0.4)	0.002	0.49 (0.29, 0.70)	0.66 (0.43, 0.89)	−0.11 (−0.46, 0.24)	<0.001
	LLN (*n*, %)	0 (0%)	0 (0%)	ns	0 (0%)	0 (0%)	0 (0%)	ns

MEF_25–75_	(% pred)	108 (103, 113)	97 (90, 104)	0.005	96 (90, 102)	114 (108, 121)	101 (90, 111)	<0.001
	z-MEF_25–75_	0.35 (0.12, 0.58)	−0.17 (−0.46, 0.11)	0.004	−0.21 (−0.45, 0.36)	0.58 (0.30, 0.86)	0.10 (−0.46, 0.67)	<0.001
	LLN (*n*, %)	1 (1.3%)	4 (7.4%)	0.158	4 (6.3%)	0 (0%)	1 (4.8%)	<0.001

FEV_1_/FVC	(% pred)	97 (95, 98)	93 (91, 95)	0.007	93 (91, 94)	98 (96, 100)	96 (93, 100)	<0.001
	z-FEV_1_/FVC	−0.37 (−0.57, −0.17)	−0.81 (−1.07, −0.55)	0,005	−0.89 (−1.12, −0.67)	−0.16 (−0.41, 0.09)	−0.45 (−0.85, −0.05)	<0.001
	LLN (*n*, %)	5 (6.3%)	10 (18.5%)	0.027	11 (17.5%)	2 (4.0%)	2 (9.5%)	0.001
FE_NO_ (ppb)^*∗*^		14.2 (10.5)	13.6 (8.7)	0.933	13.7 (9.6)	14.2 (9.0)	12.8 (12.4)	0.867

^*∗*^ median (interquartile range); ns = not significant; FEV_1_ = forced expiratory volume in one second; FVC = forced vital capacity; MEF_25–75_ = mean expiratory flow between 25 and 75% of FVC; LLN = lower limit of normal; ppb = parts per billion.

## Data Availability

The consent given by the participants does not open for sharing the full data set.
